# Evaluation of Prognosis in Cirrhotic Patients with Esophageal Variceal Bleeding Using Non-Invasive Scores

**DOI:** 10.3390/medicina61122194

**Published:** 2025-12-11

**Authors:** Murat Kırdar, Bünyamin Sarıtaş, Abdullah İlhan, Şehmus Ölmez

**Affiliations:** 1 Department of Internal Medicine, Islahiye State Hospital, 27800 Gaziantep, Turkey; muratkrdr01@gmail.com; 2 Department of Gastroenterology, Adana City Training and Research Hospital, University of Health Sciences, 01230 Adana, Turkey

**Keywords:** esophageal variceal bleeding, treatment, prognosis, CTP and MELD, non-invasive scores, King’s score

## Abstract

*Background and Objectives*: The aims of this study were to evaluate and compare the effectiveness of the Child–Turcotte–Pugh (CTP) and Model for End-Stage Liver Disease (MELD) scores, as well as the non-invasive fibrosis scores of the aspartate aminotransferase-to-platelet ratio index (APRI) and fibrosis-4 (FIB-4), the Göteborg University Cirrhosis Index (GUCI), and the King’s score, in determining the 6-week and 6-month prognoses in cirrhotic patients with EVB. *Materials and Methods*: Cirrhotic patients presenting with EVB and admitted to Adana City Training and Research Hospital between September 2017, and October 2022 were included in this study. CTP, MELD, APRI, FIB-4, King’s, and GUCI scores were recorded. The CTP stage and CTP, MELD, APRI, FIB-4, King’s, and GUCI scores were compared according to the 6-week and 6-month prognoses of the patients, and a receiver operating characteristic (ROC) analysis was performed. *Results*: The mean age of the patients was 59.4 ± 13.9 years, and 55 (64.7%) were male. The 6-week and 6-month mortality rates were 21.2% and 28.2%, respectively. The CTP, MELD, APRI, FIB-4, King’s, and GUCI scores were compared according to the 6-week and 6-month prognoses of the patients. All scores were significantly different between survivors and non-survivors (*p* < 0.05). The MELD, CTP, and King’s scores were identified as the most effective scores for predicting 6-week mortality (area under the ROC curve (AUC) of 0.888, 0.857, and 0.770, respectively). The MELD, CTP, and King’s scores were identified as the most effective scores for predicting 6-month mortality (AUC of 0.835, 0.823, and 0.734, respectively). *Conclusions*: The prognosis of cirrhotic patients presenting with EVB is poor. CTP, MELD, APRI, FIB-4, King’s, and GUCI scores were statistically significantly higher in non-survivors than in survivors. CTP and MELD scores were found to be more effective than non-invasive scores in predicting 6-week and 6-month prognoses. The most effective non-invasive score was the King’s score.

## 1. Introduction

Esophageal varices are a common and life-threatening complication of liver cirrhosis. The frequency of varices increases with liver disease severity. Esophageal variceal bleeding (EVB) is the most feared complication of varices. The risk of EVB is associated with the size of the varix, bleeding markers of the varices (fibrin plug and red wale sign), severity of cirrhosis, and hepatic venous pressure gradient. Variceal bleeding is a major cause of morbidity and mortality in patients with cirrhosis. Despite advances in diagnosis, treatment, and management, the six-week mortality of patients presenting with variceal bleeding is approximately 15–20% [1,2,3,4].

It is important to determine the prognosis of cirrhotic patients with EVB. The Child–Turcotte–Pugh (CTP) and Model for End-Stage Liver Disease (MELD) scores are the most used scoring systems for evaluating disease severity. Numerous studies have shown their effectiveness in predicting mortality associated with acute variceal bleeding [5,6,7,8,9].

Over the past few decades, various non-invasive markers, referred to as non-invasive scores, have been developed [10]. Non-invasive scores are inexpensive, reproducible, well-validated, and easily calculated using routine laboratory tests. However, they are not liver-specific and have certain limitations in conditions such as hemolysis and Gilbert’s syndrome. Their effectiveness has been demonstrated both in identifying advanced fibrosis and cirrhosis and in predicting the presence of varices and the prognosis of liver cirrhosis [10,11,12,13]. A few non-invasive scores can also be used to predict the presence of varices and the risk of bleeding [14]. Many studies have evaluated the efficacy of CTP and MELD scores, and both scores are accepted as prognostic scores; however, the prognostic value of other non-invasive scores, such as APRI and FIB-4 scores, are debated in cirrhotic patients with EVB [13,15]. The King’s and GUCI scores have not yet been evaluated.

The aims of this study were to evaluate and compare the effectiveness of these scores in determining the 6-week and 6-month prognoses in cirrhotic patients with EVB.

## 2. Materials and Methods

The study is a retrospective study which was conducted at Adana City Training and Research Hospital. Cirrhotic patients with EVB admitted between September 2017 and October 2022 were included.

The diagnosis of EVB was made in patients with upper gastrointestinal bleeding symptoms when endoscopy revealed active variceal bleeding, recent bleeding stigmata (adherent clot or fibrin plug), or fresh blood in the stomach without an alternative source of bleeding in the presence of esophageal varices.

Cirrhosis was diagnosed based on clinical, laboratory, and imaging findings or a liver biopsy. The presence of ascites was determined via a physical examination or imaging methods such as ultrasound and computed tomography (CT). Patients who were diagnosed with hepatocellular cancer (HCC) based on a histopathological examination or the presence of washout on contrast-enhanced CT or magnetic resonance imaging were recorded as having HCC [16]. HE was defined according to the West Haven criteria after excluding other potential causes of encephalopathy [17]. HRS was defined according to the International Ascites Club diagnostic criteria for hepatorenal syndrome [18].

Patient data were obtained from the hospital’s electronic database. The following data were recorded: admission date, age, sex, duration and etiology of cirrhosis, presence of complications (ascites, hepatic encephalopathy, hepatocellular carcinoma, hepatorenal syndrome, infections, and portal vein thrombosis), history of variceal bleeding, clinical presentation, time to endoscopy, medical treatments (somatostatin, octreotide, and terlipressin), endoscopic treatment (endoscopic band ligation, endoscopic sclerotherapy, and others), and the need for blood transfusion and number of blood transfusions. Laboratory parameters on admission were also evaluated. CTP, MELD, APRI, FIB-4, King’s, and GUCI scores were calculated using data obtained within the first 24 h of admission. Six-week and six-month mortality rates were also assessed.

The calculation of non-invasive scores was performed using the following formulas [19,20,21,22,23]:

MELD score:

3.78 × ln[bilirubin (mg/dL)] + 11.2 × ln[INR] + 9.57 × ln[creatinine (mg/dL)] + 6.43

APRI = [(AST/ULN)/platelet count (×10^9^/L)] × 100

FIB-4 = (Age × AST)/(Platelet count × √ALT)

GUCI Score:

[AST/Upper Limits of Normal (ULN)] × prothrombin-INR × 100)/Platelet count (×10^9^/L)

King’s score:

Age (years) × AST (IU/L) × INR/Platelet counts (×10^9^/L)

Patients with pregnancy, portosystemic shunts, bleeding from other sources (including gastric varices), an age below 18 years, no cirrhosis, and incomplete data were excluded from the study.

This study was approved by the Local Ethics Committee of Adana City Training and Research Hospital (decision number: 2022/1762) and conducted in accordance with the Declaration of Helsinki.

### Statistical Analysis

Statistical analyses were performed using the SPSS (Statistical Package for the Social Sciences) version 25.0 software package (IBM SPSS Statistics for Windows, Version 25.0. Armonk, NY, USA: IBM Corp). Categorical variables are summarized as numbers and percentages, and continuous variables are summarized as means and standard deviations (and as median and minimum–maximum values where appropriate). The Kolmogorov–Smirnov test was used to determine whether the parameters in the study were normally distributed. For normally distributed variables, the independent Student’s t-test was used. For non-normally distributed variables, the Mann–Whitney U test was used. The chi-square test was used to compare categorical variables. To evaluate prognosis, the sensitivity and specificity values of the baseline CTP, MELD, APRI, FIB-4, King’s, and GUCI scores were calculated, and the area under the receiver operating characteristic curve (AUC) was examined to determine the cut-off value. A *p*-value below 0.05 was considered statistically significant for all tests.

## 3. Results

This study included 85 patients with a mean age of 59.4 ± 13.9 years, of whom 55 (64.7%) were male. The mean CTP and MELD scores of the patients were 8.04 ± 1.9 and 13.02 ± 4.7, respectively. The mean time to endoscopy was 5.36 ± 10.3 h (range: 1–72 h). The demographic characteristics of the patients are presented in Table 1.

Both medical and endoscopic treatments administered to the patients were evaluated. The most administered medical treatment was somatostatin, and the most frequently performed endoscopic procedure was EBL. The 6-week and 6-month mortality rates were found to be 21.2% and 28.2%, respectively. The distribution of patients based on the administered medical and endoscopic therapies and mortality rate is presented in Table 2.

Laboratory values were compared according to the patients’ 6-week and 6-month prognoses. Comparisons between survivors and non-survivors are summarized in Table 3 and Table 4.

The CTP stage and CTP, MELD, APRI, FIB-4, King’s, and GUCI scores were compared according to the 6-week and 6-month prognosis of the patients. For all scores, the values were significantly higher in non-survivors than in survivors, according to the 6-week prognosis. Comparisons between survivors and non-survivors are summarized in Table 5 and Table 6.

**Figure 1:**
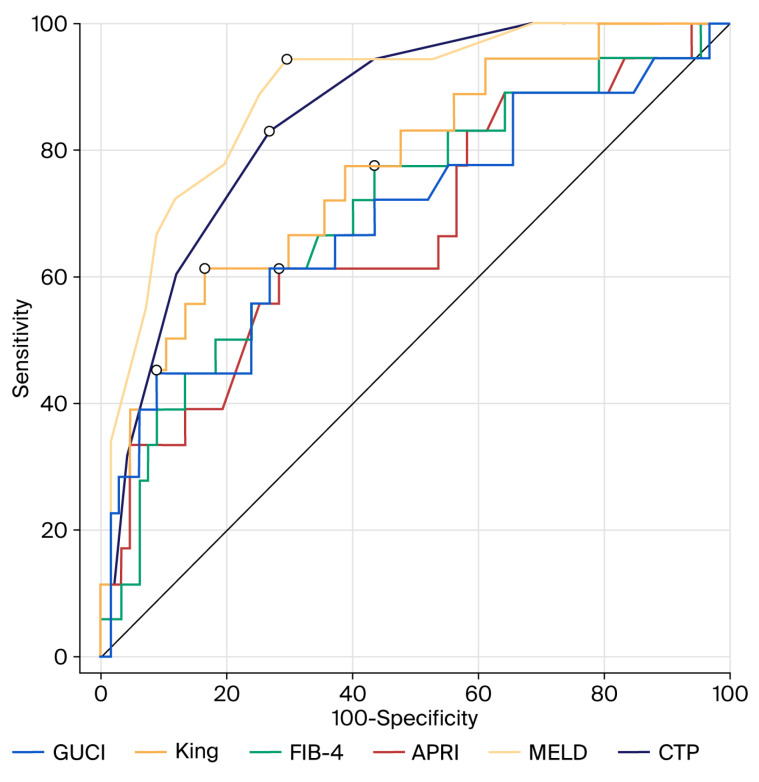
ROC curve analysis of CTP, MELD, APRI, FIB-4, King’s, and GUCI scores according to 6-week mortality outcomes.

**Figure 2:**
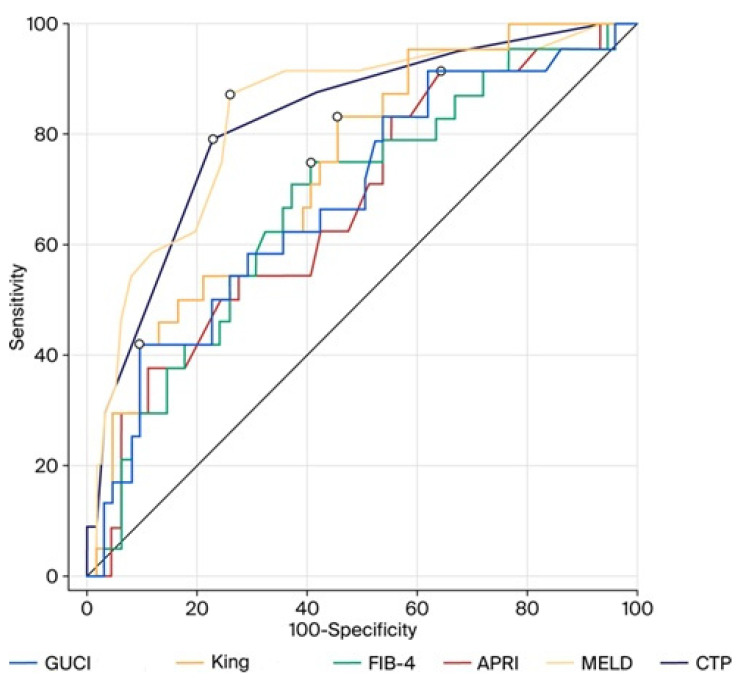
ROC analysis of CTP, MELD, APRI, FIB-4, King’s, and GUCI scores according to 6-month mortality outcomes.

The prognostic performance of non-invasive scores of 6-week and 6-month mortality were analyzed using an ROC curve analysis. Accordingly, MELD, CTP, and King’s scores were identified as the most effective scores for predicting 6-week mortality (area under the ROC curve (AUC) of 0.888, 0.857, and 0.770, respectively). According to the 6-week mortality, the cut-off (threshold) values for CTP, MELD, APRI, FIB-4, King’s, and GUCI scores are presented in Table 7. The ROC curve analysis of CTP, MELD, APRI, FIB-4, King’s, and GUCI scores according to 6-week mortality is shown in Figure 1. The ROC curve analysis of CTP, MELD, APRI, FIB-4, King’s, and GUCI scores according to 6-month mortality is shown in Figure 2. According to the 6-month mortality, the cut-off (threshold) values for CTP, MELD, APRI, FIB-4, King’s, and GUCI scores are presented in Table 8.

## 4. Discussion

Esophageal variceal bleeding is a common and important complication of liver cirrhosis [24,25]. In cirrhotic patients, evaluating the presence of esophageal varices, the risk of variceal bleeding, and the prognosis associated with variceal bleeding is important. EVB is observed in patients with advanced cirrhosis, and its prognosis is related to the severity of chronic liver disease [26]. As the number of accompanying comorbid diseases and complications increases, the prognosis worsens [27]. In our study, based on the CTP classification, 19 patients (22.4%) were classified as class A, 47 (55.2%) as class B, and 19 (22.4%) as class C. The mean MELD score was 13.02 ± 4.7, being ≤12 in 49 (57,64%) patients. The most common comorbid diseases accompanying cirrhosis were diabetes mellitus and hypertension. Ascites was present in 63 (74.1%), hepatic encephalopathy in 11 (12.9%), and hepatocellular carcinoma (HCC) in 10 (11.8%) patients; in addition, infection was present in 9 patients (10.6%).

EVB is associated with increased mortality and morbidity [28]. This leads to substantial increases in healthcare costs and represents a major public health concern [25]. EVB is a medical emergency requiring urgent and intensive intervention. The main goals of therapy are the control of active bleeding, prevention of rebleeding, and reduction in mortality. Recommendations for the management of EVB include restrictive red blood transfusion, antibiotic therapy, vasoactive drugs such as somatostatin, and bleeding control via EBL and/or sclerotherapy [1,2]. An early endoscopic intervention and the proper selection of endoscopic treatment modality improve prognosis [1,2]. The first-line endoscopic treatment of EVB is EBL [28]. In our study, the mean time to endoscopy following gastroenterology consultation after hospital admission was 5.36 ± 10.3 h (range: 1–72 h). Most patients received medical treatment with somatostatin, octreotide, or terlipressin. The most frequently applied endoscopic treatment was EBL (92.9%). Despite appropriate medical and endoscopic treatment, cirrhotic patients with EVB have a high mortality rate [24,29]. In our study, the 6-week and 6-month mortality rates were 21.2% and 28.2%, respectively.

Non-invasive scores represent simple and practical tools, and they can be easily calculated using routine laboratory tests. There are many non-invasive scoring methods, for example, the APRI, FIB-4, King’s, and GUCI scoring methods [30,31,32]. APRI and FIB-4 scores are recommended and have been proven effective in detecting the severity of liver fibrosis and cirrhosis [14,33,34]. Numerous studies have shown that non-invasive fibrosis scores are significant indicators of the presence of esophageal varices and the risk of bleeding in cirrhotic patients [12,35,36,37,38,39]. However, studies investigating their use as prognostic indicators in cirrhotic patients with EVB are limited [14].

ROC analyses are frequently used to assess the prognostic performance of prognostic scores (CTP and MELD) and non-invasive scores that are utilized to evaluate the prognosis of cirrhosis. When an ROC analysis is performed, cut-off values are determined by comparing the area under the receiver operating curves [7,40]. In general, an AUC of 0.5–0.69 suggests no to poor discrimination, 0.7–0.79 is acceptable, 0.8–0.89 is excellent, and 0.9 or more is outstanding [10]. We found in our study that, according to the ROC analysis, both the CTP and MELD scores demonstrated excellent discriminative ability in predicting both short-term (6-week) and long-term (6-month) prognoses in cirrhotic patients with variceal bleeding. In addition, the King’s and FIB-4 scores showed acceptable performance for predicting 6-week prognosis, while the APRI and GUCI scores showed poor discrimination. The King’s score maintained acceptable accuracy for long-term prognosis in these patients, while the APRI, FIB-4, and GUCI scores showed poor discrimination in long-term prognosis.

In cirrhotic patients, MELD and CTP scores are commonly used to determine prognosis. Moreover, numerous studies have demonstrated that these scores are strong predictors of short-term mortality in cirrhotic patients with EVB [41,42,43,44,45,46,47]. In our study, the MELD and CTP scores were found to strongly predict 6-week mortality in patients with EVB. The MELD score was the most accurate predictor of prognosis, with a sensitivity of 94.4% and a specificity of 70.15% (AUC: 0.888, 95% CI: 0.801–0.946, *p* < 0.001). The CTP score was as an accurate predictor, with a sensitivity of 83.33% and a specificity of 73.13% (AUC: 0.857, 95% CI: 0.764–0.923, *p* < 0.001). Similarly, 6-month mortality was best predicted by the MELD (AUC: 0.835 95% CI: 0.738–0.906) and CTP scores (AUC: 0.823, 95% CI: 0.725–0.897). Our study found that, in cirrhotic patients with EVB, the MELD and CTP scores were the most effective scores in predicting both 6-week and 6-month prognoses. These scores were superior to other non-invasive scores in determining prognosis.

Studies have been conducted on various prognostic indices in cirrhotic patients presenting with EVB. Specifically, established prognostic scores (e.g., CTP and MELD) and commonly used non-invasive fibrosis scores (e.g., APRI, and FIB-4) have been assessed to a limited degree; however, to the best of our knowledge, no studies have evaluated the utility of the King’s and GUCI scores for prognostic assessment in EVB. In our study, the APRI, FIB-4, King’s, and GUCI scores for 6-week and 6-month mortality were significantly higher in non-survivors than in survivors. The effectiveness of the non-invasive scores in predicting 6-week prognosis related to EVB ranked in the following order: the King’s score, the FIB-4 score, the GUCI, and the APRI (AUCs: 0.770, 0.704, 0.692, and 0.670, respectively). The effectiveness of the non-invasive fibrosis scores in predicting the 6-month prognosis related to EVB ranked in the following order: the King’s score, the FIB-4 score, the GUCI, and the APRI (AUCs: 0.734, 0.676, 0.681, and 0.666, respectively). The King’s score was more successful than the other non-invasive fibrosis scores in predicting both 6-week and 6-month prognoses and, thus, was identified as the most effective score for predicting 6-week and 6-month prognoses. The King’s score includes the INR value, which is a parameter that reflects the hepatic functional reserve and is also considered in the CTP and MELD scores. In the Baveno criteria, platelet count is an important factor for demonstrating the severity of portal hypertension. Low platelet counts are observed in advanced chronic liver diseases [28]. Age is another important factor that affects the prognosis of variceal bleeding [48]. For this reason, we believe that the King’s score is superior to the other scores.

The main limitations of our study are its retrospective, single-center design and the moderate patient sample size.

## 5. Conclusions

EVB is a serious complication of cirrhosis and is associated with a high mortality rate. MELD, CTP, and non-invasive scores, including APRI, FIB-4, King’s, and GUCI scores, can predict both 6-week and 6-month outcomes. In cirrhotic patients with EVB, although none of the non-invasive scores were as effective as the MELD and CTP scores in predicting either 6-week or 6-month mortality, the King’s score was found to be the most effective non-invasive score in determining the 6-week and 6-month prognoses. We believe that the King’s score represents a non-invasive score that can be used to assess the prognosis in cirrhotic patients with esophageal variceal bleeding, in addition to the MELD and CTP scores.

## Figures and Tables

**Table 1:** Demographic characteristics of patients.

Age	59.4 ± 13.9 (22–85)
Gender, *n* (%)	
Male	55 (64.7%)
Female	30 (35.3%)
Duration of cirrhosis (years)	5.12 ± 4.1 (1–25)
Accompanying diseases, *n* (%)	
Diabetes mellitus	35 (41.2%)
Hypertension	25 (29.4%)
Atherosclerotic heart disease	9 (10.6%)
Heart failure	5 (5.9%)
Etiology, *n* (%)	
HBV	23 (27.1%)
HCV	14 (16.5%)
Alcohol	11 (12.9%)
NASH	7 (8.2%)
Other	9 (10.6%)
Cryptogenic	21 (24.7%)
CTP class, *n* (%)	
A	19 (22.4%)
B	47 (55.2%)
C	19 (22.4%)
CTP score	8.04 ± 1.9 (5–13)
MELD score	13.02 ± 4.7 (6–29)
Complications, *n* (%)	
Ascites	63 (74.1%)
Encephalopathy	11 (12.9%)
HCC	10 (11.8%)
HRS	1 (1.2%)
Infections	9 (10.6%)
Portal vein thrombosis	5 (5.9%)
History of variceal bleeding, *n* (%)	27 (31.8%)
Presentations, *n* (%)	
Hematemesis	53 (62.4%)
Hematemesis and melena	11 (12.9%)
Melena	9 (10.6%)
Hematemesis + hematochezia	8 (9.4%)
Hematochezia	3 (3.5%)
Syncope	1 (1.2%)
Time to endoscopy (h) ± SD	5.36 ± 10.3 (1–72%)
Blood transfusion, *n* (%)	
Yes	66 (77.6%)
No	19 (22.4%)
Transfusion number (units) ± SD	3.30 ± 2.8 (1–17)

SD: standard deviation; min: minimum; max: maximum, CTP: Child–Turcotte–Pugh; MELD: Model for End-Stage Liver Disease; HBV: Hepatitis B Virus; HCV: Hepatitis C Virus; NASH: Nonalcoholic Steatohepatitis; HCC: hepatocellular carcinoma; HRS: hepatorenal syndrome.

**Table 2:** Distribution of patients based on administered medical and endoscopic therapies and mortality rate.

Medical Treatment	*n* (%)
Somatostatin	36 (42.3%)
Octreotide	26 (30.6%)
Terlipressin	19 (22.4%)
Untreated	4 (4.7%)
Endoscopic treatment	
EBL	79 (92.9%)
Sclerotherapy	3 (3.5%)
Combined therapy (EBL + sclerotherapy)	1 (1.2%)
Endoclips	1(1.2%)
Unable to undergo endoscopic therapy	1(1.2%)
Mortality rate	
6-week mortality	18 (21.2%)
6-month mortality	24 (28.2%)

EBL: endoscopic band ligation.

**Table 3:** Comparison of laboratory values according to patients’ 6-week prognosis.

	Survivors (*n*: 67)	Non-Survivors (*n*: 18)	*p*
WBC (/μL)	8711.6 ± 4734.5	9933.3 ± 3116.7	0.304 ^a^
Hb (gr/dL)	8.7 ± 2.1	9.2 ± 2.8	0.333 ^b^
PLT (/μL)	129.8 ± 60.2	125.2 ± 28.6	0.751 ^a^
INR	1.38 ± 0.3	1.64 ± 0.4	0.001 **^.a^
Glucose (mg/dL)	198.4 ± 105.0	201.9 ± 161.9	0.491 ^b^
Albumin (gr/dL)	2.97 ± 0.5	2.52 ± 0.5	0.001 **^.a^
Protein (gr/dL)	6.13 ± 0.8	5.76 ± 0.9	0.150 ^a^
Total bilirubin (mg/dL)	1.44 ± 1.6	3.59 ± 3.1	0.025 *^.b^
AST (U/L)	42.6 ± 27.6	91.6 ± 83.2	0.002 **^.b^
ALT (U/L)	26.6 ± 16.9	46.2 ± 40.2	0.022 **^.b^
ALP (U/L)	110.0 ± 98.9	111.9 ± 44.9	0.947 ^a^
GGT (U/L)	110.5 ± 151.8	180.6 ± 277.1	0.138 ^b^
LDH (U/L)	251.7 ± 105.1	360.4 ± 251.5	0.014 *^.b^
Urea (mg/dL)	64.3 ± 32.8	92.7 ± 83.3	0.029 *^.a^
Cr (mg/dL)	0.82 ± 0.4	1.27 ± 0.7	0.001 **^.b^
Na (mmol/L)	137.5 ± 4.2	135.1 ± 3.1	0.030 *^.a^
K (mmol/L)	4.52 ± 0.6	4.58 ± 0.7	0.788 ^b^
Lactate (mg/dL)	30.5 ± 24.1	52.6 ± 38.6	0.001 **^.b^
CRP (mg/L)	13.3 ± 25.6	32 ± 43.9	0.003 **^.b^
Procalcitonin (μg/L)	0.28 ± 0.2	4.02 ± 7.9	0.018 *^.b^

* *p* < 0.05; ** *p* < 0.001; ^a^: independent *t*-test; ^b^: Mann–Whitney U; WBC: white blood cell; Hb: hemoglobin; PLT: platelet; INR: International Normalized Ratio; AST: alanine aminotransferase; ALT: alanine aminotransferase; ALP: alkaline phosphatase; GGT: gamma glutamyl transferase; LDH: lactic dehydrogenase; Cr: creatinine; Na: sodium; K: potassium; CRP: C-reactive protein.

**Table 4:** Comparison of laboratory values according to patients’ 6-month prognosis.

	Survivors (*n*: 67)	Non-Survivors (*n*: 24)	*p*
WBC (/μL)	9006.2 ± 4856.4	8879.2 ± 3299.4	0.907 ^a^
Hb (gr/dL)	8.65 ± 2.2	9.23 ± 2.5	0.289 ^b^
PLT (/μL)	129.8 ± 61.1	126.5 ± 35.9	0.803 ^a^
INR	1.38 ± 0.3	1.57 ± 0.4	0.009 **^.a^
Glucose (mg/dL)	200 ± 108.1	196.7 ± 142.6	0.597 ^b^
Albumin (gr/dL)	2.96 ± 0.5	2.64 ± 0.5	0.009 **^.a^
Protein (gr/dL)	6.07 ± 0.8	6.04 ± 0.9	0.914 ^a^
Total bilirubin (mg/dL)	1.28 ± 1.2	3.46 ± 3.1	<0.001 **^.b^
AST (U/L)	44.2 ± 39.8	75.2 ± 62.7	0.002 **^.b^
ALT (U/L)	27.1 ± 19.3	40.2 ± 34.0	0.035 *^.b^
ALP (U/L)	93.1 ± 50.2	148.7 ± 136.7	0.020 *^.a^
GGT (U/L)	100.2 ± 143.8	187.9 ± 257.6	0.073 ^a^
LDH (U/L)	251.7 ± 07.8	330.2 ± 220.6	0.027 *^.b^
Urea (mg/dL)	65.0 ± 32.8	83.3 ± 74.7	0.122 ^a^
Cr (mg/dL)	0.84 ± 0.4	1.12 ± 0.7	0.042 *^.b^
Na (mmol/L)	137.6 ± 4.3	135.4 ± 3.1	0.025 *^.a^
K (mmol/L)	4.53 ± 0.6	4.53 ± 0.7	0.838 ^b^
Lactate (mg/dL)	31.7 ± 24.9	44.9 ± 36.7	0.060 ^b^
CRP (mg/dL)	11.9 ± 25.3	30.6 ± 39.9	<0.001 **^.b^
Procalcitonin (μg/L)	0.26 ± 0.2	3.03 ± 6.9	0.019 *^.b^

* *p* < 0.05; ** *p* < 0.001; ^a^: independent *t*-test; ^b^: Mann–Whitney U; WBC: white blood cell; Hb: hemoglobin; PLT: platelet; INR: International Normalized Ratio; AST: alanine aminotransferase; ALT: alanine aminotransferase; ALP: alkaline phosphatase; GGT: gamma glutamyl transferase; LDH: lactic dehydrogenase; Cr: creatinine; Na: sodium; K: potassium; CRP: C-reactive protein.

**Table 5:** Comparison of prognostic scores according to patients’ 6-week prognosis.

	Survivors (*n*: 67)	Non-Survivors (*n*: 18)	*p*
CTP stage *n* (%)			
CTP A	19 (28.4%)	-	<0.001 **^.a^
CTP B	40 (59.7%)	7 (38.9%)	
CTP C	8 (11.9%)	11 (61.1%)	
CTP score	7.53 ± 1.6	9.94 ± 1.6	<0.001 **^.b^
MELD score	11.6 ± 3.8	18.3 ± 4.3	<0.001 **^.b^
MELD classification			
≤12	48 (71.6%)	1(5.6%)	<0.001 ** ^a^
>12	19 (28.4%)	17 (94.4%)	
APRI	0.94 ± 0.8	1.67 ± 1.4	0.027 *^.b^
FIB-4 score	4.59 ± 3.7	6.59 ± 3.6	0.008 *^.b^
King’s score	31.6 ± 24.7	69.7 ± 49.2	<0.001 **^.b^
GUCI score	1.33 ± 1.3	2.72 ± 2.2	0.013 **^.b^

* *p* < 0.05; ** *p* < 0.001; ^a^: Chi square test; ^b^: Mann–Whitney U; CTP: Child–Turcot–Pugh; MELD: Model for End-Stage Liver Disease; APRI: aspartate transaminase-to-platelet ratio index; FIB-4: fibrosis-4.

**Table 6:** Comparison of prognostic scores according to patients’ 6-month prognosis.

	Survivors (*n*: 61)	Non-Survivors (*n*: 24)	*p*
CTP stage, *n* (%)			
CTP A	18 (29.5%)	1 (4.2%)	<0.001 **^.a^
CTP B	36 (59.0%)	11 (45.8%)	
CTP C	7 (11.5%)	12 (50.0%)	
CTP score	7.44 ± 1.5	9.58 ± 1.7	<0.001 **^.b^
MELD score	11.49 ± 3.9	16.91 ± 4.6	<0.001 **^.b^
MELD classification			
≤12	46 (75.4%)	3 (12.5%)	<0.001 ** ^a^
>12	15 (24.6%)	21 (87.5%)	
APRI	0.98 ± 0.9	1.41 ± 1.0	0.018 **^.b^
FIB-4 score	4.63 ± 3.9	6.0 ± 3.2	0.012 **^.b^
King’s score	32.8 ± 31.0	57.1 ± 38.5	0.001 *^.b^
GUCI score	1.38 ± 1.5	2.24 ± 1.8	0.010 **^.b^

* *p* < 0.05; ** *p* < 0.001; ^a^: Chi square test; ^b^: Mann–Whitney U; CTP: Child–Turcot–Pugh; MELD: Model for End-Stage Liver Disease; APRI: aspartate transaminase-to-platelet ratio index; FIB-4: fibrosis-4; GUCI: Goteborg University Cirrhosis Index.

**Table 7:** Prognostic performance of CTP, MELD, APRI, FIB-4, King’s, and GUCI scores for 6-week mortality.

	CTP	MELD	APRI	FIB-4	King’s	GUCI
AUC 95%–Cl (%)	0.857 (0.764–0.923)	0.888 (0.801–0.946)	0.670 (0.560–0.769)	0.704 (0.596–0.798)	0.770 (0.666–0.855)	0.692 (0.583–0.788)
Cut-off	8	12	0.98	3.95	51.38	2.6
Sensitivity (%) 95%-Cl (%)	83.33 (58.6–96.4)	94.44 (72.7–99.9)	61.11 (35.7–82.7)	77.78 (52.4–93.6)	61.11 (35.7–82.7)	44.44 (21.5–69.2)
Specificity 95%-Cl (%)	73.13 (60.9–83.2)	70.15 (57.7–80.7)	71.64 (59.3–82)	56.72 (44–68.8)	83.58 (72.5–91.5)	91.04 (81.5–96.6)
PPV 95%-Cl (%)	45.5 (34.8–56.5)	45.9 (36.7–55.5)	36.7 (25.4–49.6)	32.6 (25–41.1)	50 (34.2–65.8)	57.1 (34.7–77)
NPV 95%-Cl (%)	94.2 (85.2–97.9)	97.9 (87.4–99.7)	87.3 (79–92.6)	90.5 (79.6–95.9)	88.9 (81.6–93.5)	85.9 (80–90.3)
*p*	<0.001 **	<0.001 **	0.026 *	0.005 *	<0.001 **	0.014 *

* *p* < 0.05; ** *p* < 0.001; PPV: positive predictive value; NPV: negative predictive value; AUC: Area Under the ROC Curve; CTP: Child–Turcot–Pugh; MELD: Model for End-Stage Liver Disease; APRI; aspartate transaminase-to-platelet ratio index; FIB-4: fibrosis-4; GUCI: Goteborg University Cirrhosis Index.

**Table 8:** Prognostic performance of CTP, MELD, APRI, FIB-4, King’s, and GUCI scores of 6-month mortality.

	CTP	MELD	APRI	FIB-4	King’s	GUCI
AUC 95%-Cl (%)	0.823 (0.725–0.897)	0.835 (0.738–0.906)	0.666 (0.555–0.764)	0.676 (0.566–0.773)	0.734 (0.627–0.824)	0.681 (0.571–0.778)
Cut-off	8	12	0.52	3.95	22.01	2.57
Sensitivity (%) 95%-Cl (%)	79.17 (57.8–92.9)	87.50 (67.6–97.3)	91.67 (73–99)	75 (53.3–90.2)	83.33 (62.6–95.3)	41.67 (22.1–63.4)
Specificity 95%-Cl (%)	77.05 (64.5–86.8)	73.77 (60.9–84.2)	36.07 (24.2–49.4)	59.02 (45.7–71.4)	54.1 (40.8–66.9)	90.16 (79.8–96.3)
PPV 95%-Cl (%)	57.6 (45.1–69.2)	56.8 (45.6–67.2)	36.1 (31.1–41.4)	41.9 (33–51.3)	41.7 (34–49.7)	62.5 (40.5–80.3)
NPV 95%-Cl (%)	90.4 (81–95.4)	93.7 (83.7–97.8)	91.7 (73.7–97.7)	85.7 (74.4–92.5)	89.2 (76.6–95.4)	79.7 (73.5–84.8)
*p*	<0.001 **	<0.001 **	0.010 *	0.006 *	<0.001 **	0.005 **

* *p* < 0.05; ** p < 0.001; PPV: positive predictive value; NPV: negative predictive value; AUC: Area Under the ROC Curve; CTP: Child–Turcot–Pugh; MELD: Model for End-Stage Liver Disease; APRI; aspartate transaminase-to-platelet ratio index; FIB-4: fibrosis-4; GUCI: Goteborg University Cirrhosis Index.

## Data Availability

Data are available from the corresponding author upon reasonable request.

## Abbreviations

The following abbreviations are used in this manuscript:

AUC	Area Under the Receiver Operating Characteristic Curve
ALT	Alanine Aminotransferase
ALP	Alkaline Phosphatase
APRI	Aspartate Aminotransferase to Platelet Ratio Index
AST	Aspartate Aminotransferase
CT	Computed Tomography
CTP	Child–Turcotte–Pugh
Cr	Creatinine
CRP	C-Reactive Protein
DM	Diabetes Mellitus
EBL	Endoscopic Band Ligation
EVB	Esophageal Variceal Bleeding
FIB-4	Fibrosis-4
GGT	Gamma-Glutamyl Transferase
GUCI	Göteborg University Cirrhosis Index
Hb	Hemoglobin
HBV	Hepatitis B Virus
HCV	Hepatitis C Virus
HCC	Hepatocellular Carcinoma
HE	Hepatic Encephalopathy
HRS	Hepatorenal Syndrome
INR	International Normalized Ratio
LDH	Lactate Dehydrogenase
MELD	Model for End-Stage Liver Disease
Na	Sodium
NASH	Nonalcoholic Steatohepatitis
NPV	Negative Predictive Value
PPV	Positive Predictive Value
PLT	Platelet Count
ROC	Receiver Operating Characteristic
SD	Standard Deviation
ULN	Upper Limit of Normal
WBC	White Blood Cell Count
